# Surgery in congenital lung malformations: the evolution from thoracotomy to VATS, 10-year experience in a single center

**DOI:** 10.1186/s13019-021-01511-0

**Published:** 2021-05-17

**Authors:** Kenan C. Ceylan, Güntuğ Batihan, Ahmet Üçvet, Soner Gürsoy

**Affiliations:** Department of Thoracic Surgery, University of Health Sciences Dr Suat Seren Chest Diseases and Chest Surgery Training and Research Hospital Izmir, 35110, Yenişehir, Gaziler Street, 331 Izmir, Turkey

**Keywords:** Congenital lung malformation, Minimally invasive surgery, VATS, Large tumors, lung cancer, thoracotomy, video-assisted thoracoscopic surgery

## Abstract

**Background:**

Congenital lung malformation is an umbrella term and consist of various kind of parenchymal and mediastinal pathologies. Surgical resection is often required for diagnosis and curative treatment.

We aimed to review our experience in surgical treatment for congenital lung disease and present the role of minimally invasive surgery.

**Methods:**

Surgical resections performed for benign lesions of the lung and mediastinum between January 2009 and May 2019 were retrospectively analyzed. Patients who were found to have congenital lung malformation as a result of pathological examination were included in our study. Distribution characteristics of the patients according to congenital lung malformation subtypes, differences in surgical approach and postoperative results were investigated.

**Results:**

A total of 94 patients who underwent surgical resection and were diagnosed with the bronchogenic cyst, sequestration, bronchial atresia, congenital cystic adenomatoid malformation (CCAM), or enteric cyst as a result of pathological examination were included the study. There were no significant differences between pathological subtypes in the postoperative length of hospital stay and drainage duration however, perioperative complication rate was higher in the sequestration group. In addition, in the first three days postoperatively, the mean pain score was found to be lower in the VATS group compared to thoracotomy.

**Conclusions:**

Congenital lung malformations consist of a heterogeneous group of diseases and the surgical treatment in these patients can range from a simple cyst excision to pneumonectomy. Video-assisted thoracoscopic surgery should be considered as the first choice in the surgical treatment of these patients in experienced centers.

## Background

Congenital malformation of the lung is an overarching term and consists of the bronchogenic cyst, pulmonary sequestration, congenital cystic adenoid malformation, congenital lobar emphysema, bronchial atresia, and enteric duplication cyst [1, 2]. It must be emphasized that there is no consensus regarding the nomenclature for these anomalies, but “congenital lung malformations” is the term that is most widely used in the literature [3, 4]. These malformations are usually diagnosed and managed in the newborn period, in infancy, or in childhood. However, patients can also be entirely asymptomatic or present with nonspecific symptoms like cough, chest pain, or dyspnea; therefore, diagnosis may be delayed until adult age [1–3].

In literature, there are several case series of congenital lung malformations in the neonatal/early childhood period, but there isn’t enough study on the results of surgical treatment in older patients [4–6].

## Methods

### Patient selection

This retrospective study was approved by the Institutional Review Board of the Dr. Suat Seren Chest Diseases and Surgery Medical Practice and Research Center. We retrospectively reviewed medical records of patients who were diagnosed with congenital lung malformation in our clinic between January 2009 and May 2019.

Patients who underwent surgical resection and were diagnosed with the bronchogenic cyst, sequestration, bronchial atresia, congenital cystic adenomatoid malformation (CCAM), or enteric cyst as a result of pathological examination were included in our study.

Patients who radiologically compatible with congenital lung malformation but could not be diagnosed definitively in the pathological examination were excluded from the study.

Thorax computed tomography (CT), respiratory function test, and bronchoscopy were performed for all patients before surgery.

Patient demographic information, presenting symptoms, medical history, preoperative investigations, intraoperative findings, histopathology, perioperative morbidity and mortality, length of hospitalization, and drainage time were collected.

Pathological subtypes (Bronchogenic cyst, sequestration, and others) and operation procedures (VATS vs. thoracotomy) were compared in terms of postoperative length of hospital stay, drainage time, and perioperative complications. The numeric rating scale (NRS) was used for the assessment of postoperative pain, and mean scores of VATS and thoracotomy groups were compared.

A perioperative complication was defined as any complication occurring within 30 days after surgery. Prolonged air leakage was defined as an air leak more than 7 days after surgery. Postoperative mortality was defined as death occurring within 30 days post-surgery.

### Surgical management

All patients were placed in the lateral decubitus position under general anesthesia with selective one-lung ventilation.

VATS was performed through two or three incisions. Camera port was located at the 7th or the 8th intercostal space on the mid-axillary. The utility incision was located at the 4th of 5th intercostal space on the mid-axillary line. The third port was opened on the posterior axillary line if needed. The soft tissue retractor was used at the utility incision and the rigid trocar was used for the camera port. The described port locations were varied depending on lesion location and the surgeon’s personal preference.

A 10–15 cm posterolateral skin incision was made, and serratus anterior muscle was spared for patients who underwent thoracotomy. The thoracic cavity was entered through the 4th, 5th, or 6th intercostal space preserving the intercostal nerve. There was no need for rib cutting or resection in our cases. In rare cases, rib fractures occurred with the effect of the retractor during the operation. These fractures were fixed at the end of the operation and an intercostal nerve blockade was applied. In patients who underwent thoracotomy, the transcostal suture technique was used to reduce postoperative pain while approximating the ribs.

The choice of the type of surgical procedure was the personal preference of the surgeon, but some patient-related factors were considered in this choice, also.

Thoracotomy was preferred in the presence of previous pulmonary infections, pleural thickening, cystic lesions larger than 5 cm, and the inability to tolerate single lung ventilation. However, with the increased experience, VATS for congenital lung malformation has increased relative to thoracotomy. Therefore, the presence of the above-mentioned conditions should not be considered strictly contraindicated.

Mediastinoscopy was also performed in one patient with the mediastinal cystic lesion, and total excision was achieved.

Lobectomy, pneumonectomy, wedge resection, or simple excision were performed, and intra-operative frozen section analysis was worked on the specimen if needed in case of suspicion of any malignancy.

### Statistical analysis

Data were analyzed using SPSS 22.0 (SPSS Inc., Chicago, IL, USA). Descriptive statistics for categorical variables are reported as frequency and percentage, and continuous variables are reported as mean value ± standard deviation (SD) or median (range) as appropriate.

Categoric variables were analyzed using Pearson’s chi-square test or Fisher exact test. All statistical tests were two-sided, with p less than 0.05 defined as achieving statistical significance.

## Results

From January 2009 to May 2019, a total of 94 patients fitted the criteria for inclusion in this retrospective study. There were 43 men and 51 women. The median age was 41, with a range of 16 to 78 years (Table 1). 46(%48.9) of 94 patients were asymptomatic, and the most common symptom was chest pain (%15.9) (Table 2).

**Table 1:** Patients characteristics

Variables	Value
**Median age (range)**	**41(16–78)**
**Sex, n (%)**	
➢ Male	43(45.7)
➢ Female	51(54.3)
**Pathology, n (%)**	
➢ Bronchogenic cyst	71(75.5)
• Mediastinal	45(47.8)
• Parenchimal	26(27.6)
➢ Pulmonary sequestration	15(15.9)
• Intralobar	11(11.7)
• Extralobar	4(4.2)
➢ Enteric duplication cyst	4(4.2)
➢ Bronchial atresia	2(2.1)
➢ Congenital cystic adenomatoid malformation	2(2.1)
**Location of malformation, n (%)**	
➢ Mediastinal	47(50.0)
➢ Parenchymal	47(50.0)
**Surgical approach, n (%)**	
➢ Thoracotomy	67(71.2)
➢ VATS	26(27.6)
➢ Mediastinoscopy	1(1.1)

**Table 2:** Presenting symptoms

Symptom	N (%)
Chest pain	15(15.9)
Cough	11(11.7)
Dyspnea	10(10.6)
Hemoptysis	9(9.6)
Fever	2(2.1)
Asymptomatic	47(50.0)

The most common types of operation were cyst excision (*n* = 42, %44.6) and lobectomy (*n* = 30, %31.9). Intraoperative complications were seen in 3 (%3.2) cases.

The median postoperative length of stay and drainage duration were both five days. Perioperative complications were seen in 13 (%13.8) patients. The most seen perioperative complication was prolonged air leakage (*n* = 11, %11.7). There was no intraoperative or perioperative death (Table 3).

**Table 3:** Perioperative findings

Variables	Value
**Operation, n (%)**	
➢ Cyst excision	42(44.7)
➢ Lobectomy	30(31.9)
➢ Wedge resection	13(13.8)
➢ Segmentectomy	8(8.5)
➢ Pneumonectomy	1(1.1)
**Postoperative length of hospital stays, day, median (range)**	5(2–10)
**Drainage duration, day, median (range)**	5(1–18)
**Intraoperative complication, n**	
➢ Esophageal laceration	2
➢ Bronchial laceration	1
➢ Total (%)	3(3.2)
**Perioperative complication, n (%)**	
➢ Prolonged air leakage	11(11.7)
➢ Hemorrhage	1(1.1)
➢ Pneumonia	1(1.1)
➢ Total	13(13.8)

The most common type of lung malformation was a bronchogenic cyst (*n* = 71, %75.5).

There were no significant differences between pathological subtypes in the postoperative length of hospital stay and drainage duration. However, in cases of sequestration, the perioperative complication rate was significantly higher (*p* = 0.01) (Table 4).

**Table 4:** Comparison of perioperative and postoperative results between pathological subtypes and surgical techniques

Pathological subtypes	Postoperative length of hospital stays, day, median (***p*** = 0.843)	Drainage duration, day, median (***p*** = 0.459)	Perioperative complication, n (%) (***p*** = 0.022)
**Bronchogenic cyst (*n*** **= 71)**	5.59	5.07	5 (7.2)
**Sequestration (*n*** **= 15)**	6.00	5.54	5 (38.5)
**Others (*n*** **= 8)** (Enteric duplication cyst, bronchial atresia, congenital cystic adenomatoid malformation)	6.30	5.40	2 (20)
**Operation type**	**Postoperative length of hospital stays, day, median(*p*** **= 0.706)**	**Drainage duration, day, median(*p*** **= 0.717)**	**Perioperative complication, n (%) (*p*** **= 0.343)**
**VATS**	6.04	5.38	5(19.2)
**Thoracotomy**	5.74	5.12	7(12.3)
**Postoperative period (day)**	**Pain score in VATS group (mean ± SD)**	**Pain score in Thoracotomy group (mean ± SD)**	***P*** **value**
**1**	3.23 ± 0.84	3.86 ± 0.92	0.045
**2**	2.47 ± 0.80	3.15 ± 1.15	0.025
**3**	2.45 ± 0.85	3.25 ± 1.25	0.025

The mean postoperative length of hospital stay, and drainage duration was longer in the VATS group, but this data wasn’t statistically significant (*p* = 0.706/*p* = 0.717). There wasn’t a significant difference between the VATS and thoracotomy group in perioperative complications (Table 4).

The mean pain scores were lower in the VATS group in the first three days of the postoperative period, and this difference was statistically significant (Table 4).

## Discussion

The spectrum of congenital lung malformations includes bronchogenic cyst, sequestration, congenital cystic adenomatoid malformation (CCAM), bronchial atresia, enteric, and duplication cysts.

Although congenital lung malformations are usually detected antenatally, the diagnosis may be missed until later in life, especially in asymptomatic cases.

The respiratory system begins to develop from the ventral wall of the primitive foregut at about the 4th week of gestation. This process continues until early childhood and consists of five stages: Embryonic, pseudoglandular, canalicular, saccular, and alveolar. Most of the congenital lung malformations develop in the pseudoglandular phase, which is completed at the end of the 16th week [7, 8].

Despite some similarities, congenital lung malformations are a heterogeneous group and cover a wide range of disorders. Therefore, we discussed the types of malformations in our case series under separate headings.

### Bronchogenic cysts

A bronchogenic cyst is the most common congenital malformation of the mediastinum [9, 10]. They usually present as small, solitary cysts located in the mediastinum or pulmonary parenchyma. In imaging techniques, the density/intensity of the bronchogenic cysts may vary according to the cyst content, and they can mimic other mediastinal or parenchymal pathologies [11, 12]. Therefore, the bronchogenic cyst should be considered in the differential diagnosis of mediastinal and parenchymal lesions. Peripheral bronchogenic cysts are usually seen as multiloculated, thin-walled parenchymal lesions. They tend to have bronchial communication, and this situation can cause recurrent pneumonia, infection of the cyst, fever, sepsis, respiratory distress, or even severe hemoptysis [12, 13]. It was also reported that malignancy could develop on the wall of long-standing bronchogenic cysts. Therefore, the consensus in the treatment of this congenital malformation is complete surgical resection, even if the patient is asymptomatic [9–13].

In our experience, we detected two cases of malignant tumors arising from the bronchogenic cyst. One of them was mucoepidermoid carcinoma, and the other one was atypical carcinoid tumors of the lung. In both cases, bronchogenic cysts were located in pulmonary parenchyma and required anatomic lung resection. We also detected bronchogenic cyst incidentally in two cases of non-small cell lung cancer intraoperatively. In these two cases, the tumor was centrally located and accompanied by multiple mediastinal lymph nodes. The bronchogenic cyst was also located in the mediastinum and resected with other mediastinal lymph nodes. This is important for confusing the surgeon by creating the impression of a lymph node invading the bronchus. Therefore, in such cases, the result of the frozen section should be waited before beginning the anatomic resection.

**Fig. 1:**
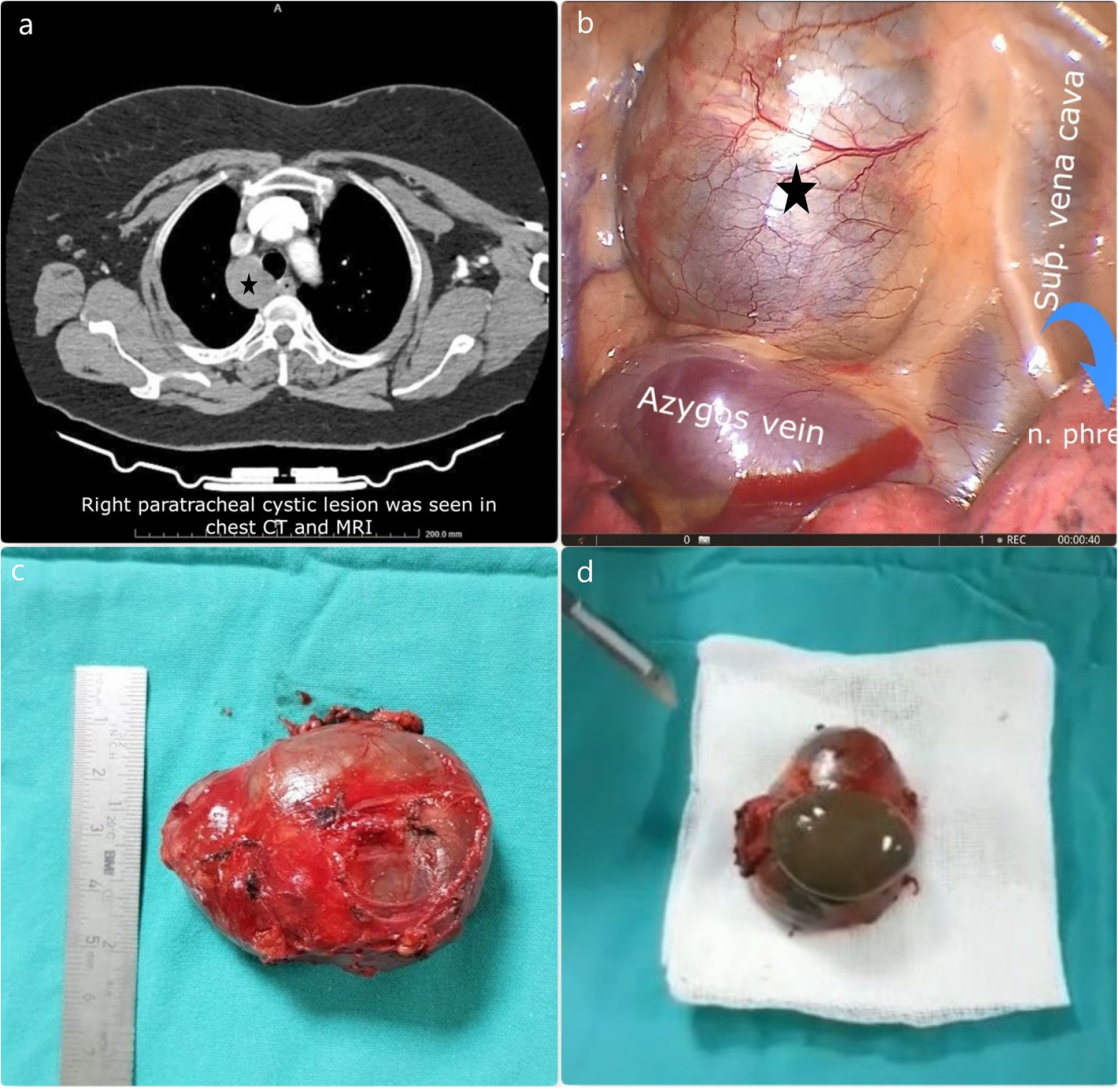
The right mediastinal cystic lesion was observed in the thorax CT (Fig. 1**a**). VATS resection of the mediastinal bronchogenic cyst (Asterisk) was performed. Brown mucoid content was observed in the macroscopy of the resection material (Fig. 1**d**)

We retrospectively analyzed 71 cases of a bronchogenic cyst in our series. Bronchogenic cysts were located in the mediastinum in 45 and pulmonary parenchyma in 26 out of 71 cases (Fig. 1). VATS procedure was performed in 16 out of 71 patients. The intraoperative complication was seen in two cases. In the first case, esophageal and bronchial laceration were seen together. In the other case, only esophagus perforation was seen. In these cases, the wall of the bronchogenic cyst strongly adhered to the bronchus and esophagus. Both lacerations occurred while dissection of the cyst wall from surrounding structures. These complications were detected intraoperatively and repaired without any problem.

**Fig. 2:**
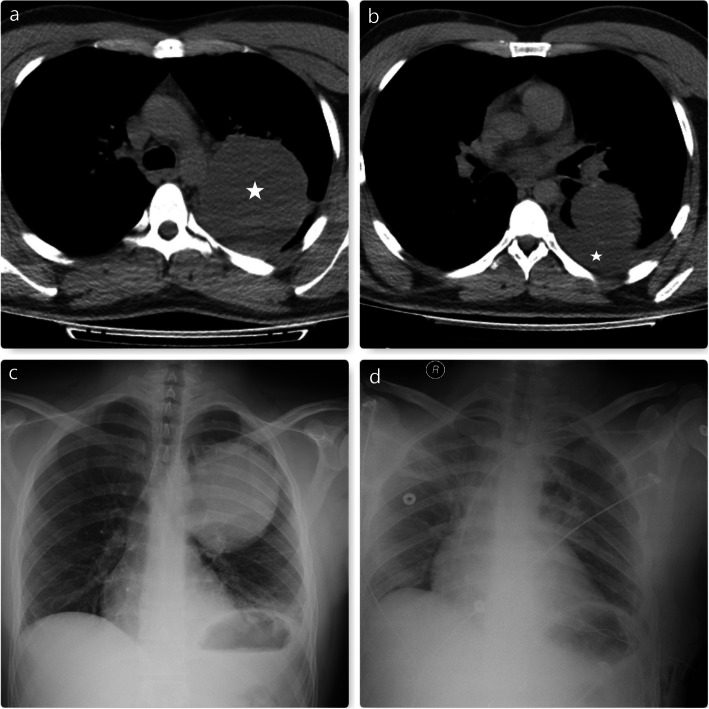
**a**. The left-sided giant bronchogenic cyst is seen in the thorax CT (Asterisk). **b**. This image shows pleural effusion accompanying bronchogenic cyst (Asterisk). Such effusions may occur due to atelectasis and disruption of lymphatic drainage, or they may be indicative of perforation in the cyst. In this case, perforation was detected intraoperatively. **c** and **d** indicate preoperative and postoperative chest radiograms

Bronchogenic cysts may be infected, and this may cause an increase in cyst pressure and perforation [11]. In the case of perforation, the infected material may spread to the pleural cavity or mediastinum and cause a serious infection that can progress to sepsis. Therefore, surgical intervention should be planned immediately in cases with suspected perforation. We have observed bronchogenic cyst perforation in one of our patients and surgical resection was performed (Fig. 2).

### Sequestration

**Fig. 3:**
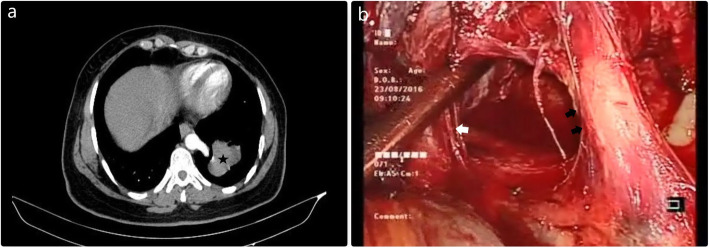
Extralobar sequestration (Asterisk) was seen in the left lower lobe, and the arterial supply of the pulmonary sequestration was detected preoperatively via the thorax CT (Fig. 3**a**). VATS resection was performed. Arterial blood supply (Black arrows) from the aorta was seen intraoperatively and dissected with an endoscopic vascular stapler (Fig. 3**b**). The white arrow indicates the pulmonary vein

Pulmonary sequestration is a rare malformation characterized by non-functional parenchymal mass, which receives arterial supply from aberrant systemic arteries [14, 15]. Pulmonary sequestration is divided into intralobar sequestration, which is located in normal pulmonary parenchyma without its pleural covering, and extralobar sequestration, which is separated from normal lung tissue by its pleural covering. Because of its distinctive arterial supply, this congenital anomaly may also be considered as a vascular malformation [15, 16]. The arterial supply of the pulmonary sequestration is usually from the descending thoracic or abdominal aorta, and venous drainage is usually toward the pulmonary veins (Fig. 3).

Patients can remain asymptomatic and be diagnosed incidentally or presented with dyspnea, cyanosis, recurrent pulmonary infection, and hemoptysis. Malignant transformation may also be seen in these patients. Therefore, surgical resection is the main treatment modality for pulmonary sequestrations, even for an asymptomatic patient [14–16].

In our series, we reviewed 15 patients with pulmonary sequestration. 11 of 15 patients had intralobar sequestration, and 4 had extralobar sequestration. VATS procedure was performed successfully in 6 out of 15 patients without any intraoperative complications. We also performed pneumonectomy in one case with synchronous extralobar pulmonary sequestration and centrally located lung cancer.

**Fig. 4:**
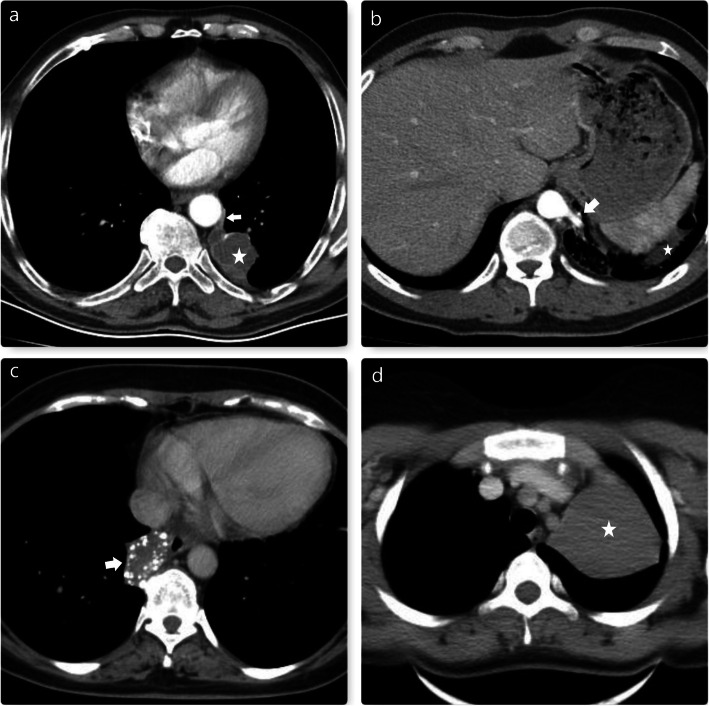
**a** and **b** show left-sided intralobar sequestrations (Asterisks). Arrows indicate aortic branches. **c**. Arrow indicates right-sided extralobar sequestration. Rarely, these lesions may contain extensive calcifications. **d**. The asterisk indicates left-sided extralobar sequestration

Preoperative evaluation of the arterial supply of pulmonary sequestration is essential for reducing the risk of intraoperative vascular injury [17, 18]. Therefore, we used 3D multidetector CT angiography in all cases of pulmonary sequestration preoperatively. Thorax CT images of some of our cases are seen in Fig. 4.

Compared to other congenital lung malformations, patients with sequestration had a higher rate of perioperative complications (%40.0), especially prolonged air leakage, and this data was statistically significant (*p* = 0.01). This result can be explained by the need for anatomic lung resection instead of simple cystectomy in patients with pulmonary sequestration.

### Congenital cystic adenomatoid malformation (CCAM)

CCAM is a rare malformation characterized by cystic areas in the lung parenchyma and adenomatous overgrowth of the terminal bronchioles [19]. Most of the cases are diagnosed prenatal or postnatally, and approximately 40% of the cases present with hydrops fetalis [20]. In the presence of a small localized lesion, patients can remain asymptomatic for a long time and can be detected incidentally in later years, but most of the patients present with unresolving pulmonary infiltration, recurrent pneumonia, pneumothorax, or pleural effusion [19, 20].

Surgical resection is the main treatment in patients with CCAM. The size and extent of the lesion determine the extent of surgical resection [3, 4, 8, 21].

**Fig. 5:**
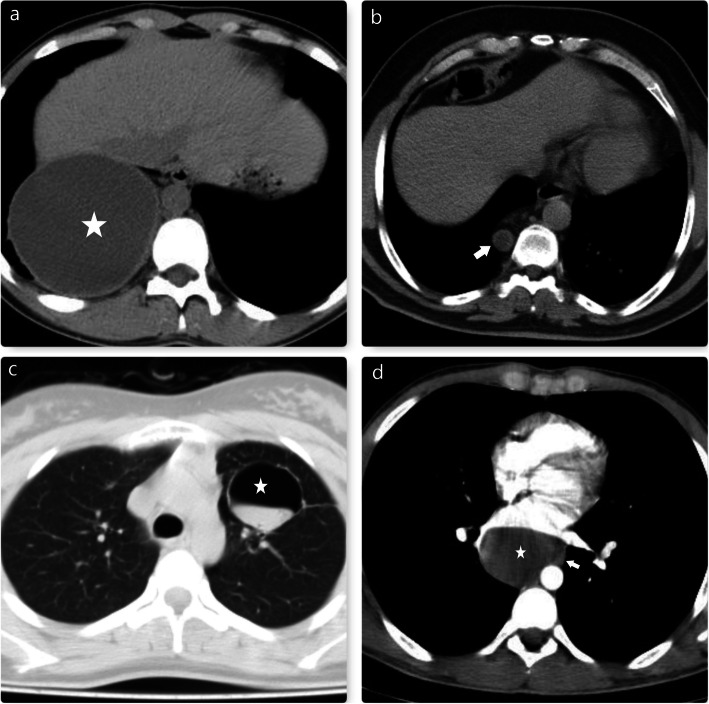
**a**. Chest CT indicates the right-sided giant enteric cyst (Asterisk). In this case, we preferred open surgery to performed complete resection. **b**. A much smaller enteric cyst is observed than in the previous case (Arrow). Therefore, we have preferred VATS for the excision of this cystic lesion. **c**. Thorax CT image of the left-sided congenital cystic adenomatoid malformation is seen (Asterisk). The left upper lobectomy was performed. **d**. This thorax CT belongs to the patient with an esophagus duplication cyst (Asterisk). Arrow indicates the wall of the esophagus. There is no contraindication, but these areas of the mediastinum are challenging localizations for the VATS procedure. Thus, we preferred open surgery in this case

In our series, we surgically treated 2 cases of CCAM. One of these two patients had dyspnea, and the other had mild hemoptysis. Left upper and left lower lobectomy was needed to complete resection (Fig. 5).

### Bronchial atresia

Bronchial atresia is a rare congenital malformation characterized by focal interruption of lobar, segmental, or subsegmental bronchus [22].

In the embryological process, bronchial buds occur at the end of the 4th gestational week and continue to branch between the 7th–16th weeks [7]. Defect in vascularization can impact the branching of the bronchial buds and can cause interruption of a lobar or segmental bronchus. This results in mucus impaction and distension of the corresponding pulmonary parenchyma. Like many other malformations, bronchial atresia is most often diagnosed in the neonatal period or the young child. Surgical resection must be considered to make a diagnosis and prevent possible complications, even in asymptomatic patients [21, 22].

In our series, bronchial atresia was detected in 2 cases. In these cases, atresia was containing left upper lobe anterior, and apicoposterior segment bronchus and pulmonary parenchyma distal to these bronchi were hyperinflated. Videothoracoscopic left upper lobe tri-segmentectomy was performed without any peri or postoperative complication in both cases.

### Enteric duplication cyst

Enteric duplication cyst is a rare congenital malformation that originate from any part of the gastrointestinal tract [1, 2]. It was reported that approximately 20% of cases are located in the mediastinum, and most of them are detected in childhood. Symptoms depend on the size and location of the cyst, but dyspnea, stridor, cough, and chest pain may be present [23]. In our series, we detected 4 cases of mediastinal enteric duplication cysts, and surgical excision was performed.

Today VATS procedure is widely used in the diagnosis and treatment of different kinds of pulmonary, mediastinal, and pleural pathologies, and its indication is extending day by day.

In several studies, VATS was compared with thoracotomy, and it has been described as a safe and feasible technique for the treatment of congenital lung diseases (Table 5).

**Table 5:** Literature review

Author	Year	Number of Patients	Patient group	Key results
Jung HS. et al. [24]	2014	113 (All patients underwent VATS; 4 patients were converted to open thoracotomy)	Bronchogenic cyst	The median operation time was 96.8 min (range, 15–320 min). There were no operative mortalities or major postoperative complications. VATS excision of bronchogenic cysts described as safe and feasible.
Guo et al. [25]	2016	99 (V:65, T:34)	Bronchogenic cyst	The VATS group had shorter operative time (108.77 ± 47.81 vs. 144.62 ± 55.16, *P* = 0.001), shorter hospital stay and drainage time (4.94 ± 2.01 vs. 8.64 ± 5.52 days, *P* = 0.001; 2.52 ± 1.29 vs. 3.71 ± 1.55 days, *P* < 0.001, respectively).
Wang et al. [26]	2018	119 (All patients underwent VATS; 1 patient was converted to open thoracotomy)	Bronchogenic cyst	Mean operative time was 103.8 ± 41.6 min (40–360 min). The intraoperative complication rate was 3.4%. VATS was described as safe and reliable for the management of MBCs
Liu et al. [27]	2013	42 (V:18, T:24)	Pulmonary sequestration	No significant differences were found between VATS and thoracotomy group in terms of the duration of operation, blood loss, amount of chest drainage, duration of chest drainage, length of postoperative hospital stay, and complications.
Sun X. et al. [28]	2014	69 (V:9, T:55)	Pulmonary sequestration	Length of postoperative hospital stay was shorter in VATS group both for ILS and ELS patients. (6.6 ± 1.5 vs 9.1 ± 3.2 days, *P* = 0.001) (7.2 ± 0.84 vs 9.2 ± 3.2 days, *p* = 0.002
Vu et al. [29]	2008	36 (V:12, T:24)	CCAM	Patients in the VATS group had significantly longer operative time (mean difference of 61.3 min; 95% confidence interval [CI], 30.5–92.1) but shorter postoperative hospital stay (mean difference of 5.7 days; 95% CI, 0.9–10.4) and duration of tube thoracostomy (mean difference of 2.6 days; 95% CI, 0.7–4.5)
Makhija Z. el al [30].	2011	102 (V:70, T:21, Others:11^*^)	Congenital cystic lung malformations	There isn’t any comparison between VATS and thoracotomy in this study, but it was reported that pneumonia and bronchogenic cysts predictors of the need for a more extensive pulmonary resection.
Current study		94 (V:26, T:67, M: 1)	Congenital lung malformations	No significant differences between VATS and thoracotomy group in terms of mean postoperative length of hospital stay, drainage duration and perioperative complications (6.04 vs 5.74 days, *p* = 0.706) (5.38 vs 5.12 days, *p* = 0.717) (% 19.2 vs % 12.3, *p* = 0.343)

V: VATS, T: Thoracotomy, M: Mediastinoscopy, MBC: Mediastinal bronchogenic cyst, CCAM: Congenital cystic adenoid malformation ILS: Intralober sequestration, ELC: Extralober sequestration

^*^Sternotomy: 5, Mediastinoscopy: 4, laparoscopy: 1 and cervical: 1

In addition to the described benefits, VATS also allows excellent exposure of the entire hemithorax and allows the entire surgical team to follow every stage of the operation, but this procedure should be encouraged only for experienced surgeons.

With the increasing experience in our center, VATS has become the increasingly preferred method of surgical approach in patients with congenital lung malformation.

In our series, we performed a VATS procedure in 26 out of 94 patients. The intraoperative complication was seen in one case (esophagus laceration), and perioperative complication was seen in 5 cases (%19.2).

There weren’t any statistically significant differences between VATS and thoracotomy groups in terms of perioperative complications, median postoperative length of hospital stays, and median drainage duration but the mean postoperative pain score was significantly lower in the VATS group.

This study had some limitations. First, it is a retrospective study, and patient selection bias existed. Second, congenital lung malformations consist of a heterogeneous group of patients; therefore, it is difficult to make comprehensive inferences. Third, the criteria for selecting VATS were not defined clearly, and we analyzed only a small number of patients at a single institution, a further large-scale prospective study will be necessary to investigate the efficacy and feasibility of VATS.

## Conclusions

In conclusion, congenital lung malformation is an umbrella term and consist of various kind of parenchymal and mediastinal pathologies. To prevent future complications, surgery is always almost needed, even in asymptomatic patients.

In this study, there are no significant differences between VATS and thoracotomy groups in terms of intraoperative and perioperative complication rates, drainage, and hospitalization durations, but the advantage of VATS in postoperative pain control was also seen in our study. Because of the possible and described benefits of minimally invasive surgical techniques, VATS should be considered as the first choice in the surgical treatment of patients with congenital lung malformations.

## Data Availability

Not applicable.

## Abbreviations

CCAM: Congenital Cystic Adenomatoid Malformation
CT: Computed Tomography
NRS: Numeric Rating Scale
SD: Standard Deviation
VATS: Video-Assisted Thoracic Surgery
